# Dengue and Hypokalemic Paralysis: A Rare Association

**DOI:** 10.7759/cureus.44705

**Published:** 2023-09-05

**Authors:** Ujala Akhtar, Muhammad Hamza Mushtaq, Sarah Nisar, Awais Naeem, Fahad Naim

**Affiliations:** 1 Medicine, Khyber Teaching Hospital, Peshawar, PAK; 2 Internal Medicine, Lady Reading Hospital, Peshawar, PAK; 3 Internal Medicine, Khyber Medical College, Peshawar, PAK; 4 Internal Medicine, Khyber Teaching Hospital, Peshawar, PAK

**Keywords:** acute hypokalemic paralysis, dengue induced motor weakness, dengue fever/complications, hypokalemia, acute motor weakness

## Abstract

Dengue is one of the most common mosquito-borne viral illnesses in tropical areas, including Pakistan. Presentation varies from a self-limiting flu-like illness to life-threatening conditions like hemorrhagic shock and multi-organ dysfunction leading to death. In the absence of vomiting and diarrhea, electrolyte abnormalities are rare findings. Though Guillain-Barré syndrome is a known association of viral illnesses presenting with flaccid paralysis, there is a possibility for dengue to cause hypokalemia without apparent gut or renal losses. Dengue-associated hypokalemic paralysis is an underrecognized entity but has a favorable outcome. The clinician should suspect this in patients presenting with motor weakness in dengue-endemic areas. Neurological complications of dengue are reported frequently now, so early recognition of these neurological manifestations is needed for the successful recovery of patients. Here, we discuss a case of dengue-induced hypokalemia presenting with acute flaccid paralysis.

## Introduction

Dengue is one of the most common mosquito-borne viral illnesses in tropical areas, including Pakistan. The presentation of dengue varies from a self-limiting flu-like illness to life-threatening conditions like hemorrhagic shock and multi-organ dysfunction leading to death [1]. Neurological complications of dengue are very rare (4%) [2] and include encephalitis, Guillain-Barré syndrome, myositis, optic neuritis, and hypokalemic paralysis. Among these, hypokalemic paralysis is very rare [3,4] and has an uncertain pathogenesis [5].

Dengue-associated hypokalemic paralysis is transient and resolves completely with potassium replacement. It can present as pure motor weakness of all four limbs accompanied by areflexia or hyporeflexia. We present a case of a 30-year-old lady already diagnosed with dengue fever who presented to us with lower limb weakness.

## Case presentation

A 30-year-old patient with no known co-morbidities diagnosed with a history of high-grade fever, headache, and myalgias for the previous three days presented to the emergency department of a tertiary care hospital with complaints of lower limb weakness for one day.

Her vitals on admission were BP 100/70 mmHg (standing), pulse 108/min, temperature 38° C, and respiratory rate 16/min.

She denied a history of diarrhea, vomiting, respiratory problems, or any urinary symptoms. There was no prior history of recent vigorous exercise, thyroid disease, or heavy carbohydrate meals. Her medical history and family history were insignificant for any such weakness.

On neurological examination, GCS was 15/15, and cranial nerves and upper limb sensory-motor examination were normal. On lower limb examination, there were absent deep tendon (ankle, knee) reflexes and 2/5 power with down-going planters.

The rest of her systemic examination, including respiratory, gastrointestinal, and cardiovascular, was unremarkable.

**Figure 1:**
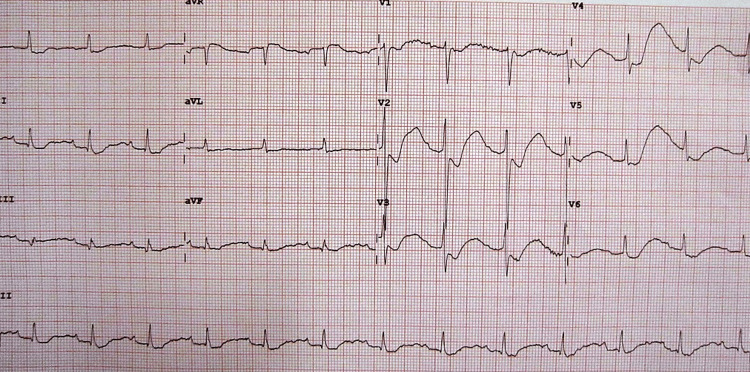
ECG of the patient before potassium chloride infusion showing ST segment depression, U wave, and prolonged QU interval

On the day of admission, the laboratory tests revealed normal platelet counts and a decreased leukocyte count with lymphopenia. Additionally, the serum urea, creatinine, urine R/E, and glucose were within normal limits. Initially, her serum electrolytes were normal, except for low potassium. Her serum electrolytes were monitored daily during treatment. Serum potassium was 2.8 mmol/L on day 2 and 3.6 mmol/L on day 3. Her ABGs were normal. All baseline investigations on admission are given in Table 1. An electrocardiogram (ECG) on admission showed changes that were consistent with hypokalemia and included ST segment depression, significant U wave, and prolonged QU interval (Figure 1).

**Table 1:** Laboratory test results of the patient g: gram, mg: milligram, pg: picogram, L: liter, dL: deciliter, µL: microliter, mmol: millimole, L: liter, U: units.

Laboratory parameter	Result	Reference range	Laboratory parameter	Result	Reference range
WBC	2.7	4-11 10^3/*uL*	Creatinine	0.56	0.42-1.06 mg/dL
Hemoglobin	10.2	11.5-17.5g/dL	Sodium	139	135–150 mmol/L
Platelets	119	150-450 10^3/*uL*	Potassium	2.57	3.5-5.1 mmol/L
Neutrophils	81.5%	40-75%	Chloride	112.7	96-112mmol/L
Lymphocytes	13.3%	20-45%	Magnesium	1.8	1.5-2.5mg/dL
Creatine kinase	434	26-140U/L	HbsAg	Negative	
Alt	162	10-50 U/L	Anti-HCV	Negative	
Alkaline phosphatase	119	35-104U/L	RBS	139	70-140mg/dL
Total Bilirubin	0.63	0.1-1.0mg/dL	Dengue NS1	Positive	
S. Urea	8.6	10-50mg/dL			

She was diagnosed with acute dengue fever with hypokalemia and started on appropriate supportive treatment, aiming to correct the electrolyte abnormality. Her net potassium deficit was calculated, and intravenous potassium chloride (KCL) was given through a peripheral line at a rate of 10 mmol/hr. On the correction of her serum potassium to 3.5 mg/dl, the patient regained strength in her lower limbs with a power of 5/5 on examination and reflexes of the lower limbs.

**Figure 2:**
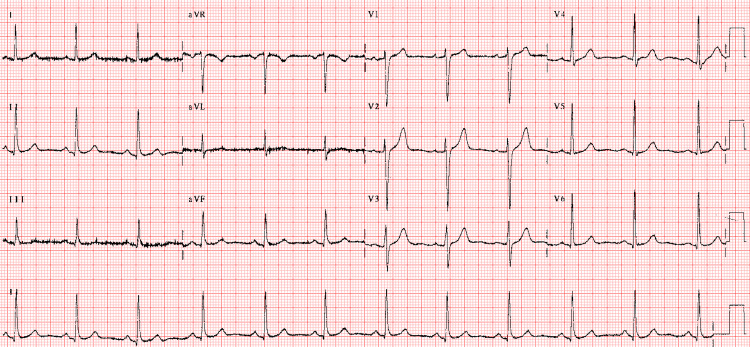
ECG after potassium chloride replacement

After intravenous KCL replacement, an ECG was repeated, and that came to normal Figure 2.

## Discussion

Dengue has been regarded as a nonneurotropic virus [6]; however, rare instances of neurological complications have been found, which include encephalopathy, GBS, myositis, meningitis, and acute pure motor weakness [7]. The pathogenesis of neurological manifestations includes the neurotrophic effect of the dengue virus, related to the systemic effects of dengue infection, and immune-mediated [8]. Serotypes commonly associated with neurological manifestations are DENV-2 and DENV-3 [9].

There is less literature available showing the association between acute motor weakness and dengue fever. Hira et al. concluded in their research that dengue virus infection led to acute neuromuscular weakness because of hypokalemia, myositis, and Guillain-Barré syndrome [10]. GBS had also been reported [11-13]. Sanjeev et al. reported a case series of three confirmed dengue patients presenting with acute reversible motor weakness due to hypokalemia [14]. Roy et al. also reported two cases of dengue patients with reversible motor weakness due to hypokalemia [15]. A review of the published literature reveals that 35 instances of hypokalemic paralysis associated with dengue have been recorded on the Indian subcontinent [16].

Given that there were no apparent renal or gastrointestinal causes of potassium depletion or history of previous similar episodes and complete resolution of symptoms on potassium replacement [17], a diagnosis of dengue-associated hypokalemia was the most appropriate differential for this case.

The exact mechanism of hypokalemia in dengue is unclear, but there is a certain hypothesis that it can be due to a transcellular shift in response to insulin release or self-limiting transient tubular dysfunction leading to potassium excretion [18]. Further study is needed in this regard and needs to be published, as dengue is on a rising trend and neurological complications are reported frequently now [19,20]. Physicians in endemic areas need to open their minds to unusual presentations like hypokalemic paralysis, which could be life-threatening but easily treatable.

## Conclusions

Dengue-associated hypokalemic paralysis is an underrecognized entity but has a favorable outcome. The clinician should suspect this in patients presenting with motor weakness in dengue-endemic areas. Neurological complications of dengue are reported frequently now, so early recognition of these neurological manifestations is needed for the successful recovery of patients. Further study is needed in this regard and needs to be published, as dengue is on the rise. Dengue-induced hypokalemia should be kept under differential diagnosis and should be ruled out, especially in endemic countries during dengue outbreaks and in cases where the etiology is uncertain. A high degree of suspicion in endemic areas can help in picking up more cases, thereby helping in understanding the true extent of neurological complications in dengue fever. 
